# Serum 25-hydroxyvitamin D levels in patients with Granulomatosis with Polyangiitis: association with respiratory infection

**DOI:** 10.6061/clinics/2017(12)02

**Published:** 2017-12

**Authors:** Mariana O. Perez, Ricardo M. Oliveira, Mauricio Levy-Neto, Valeria F. Caparbo, Rosa M.R. Pereira

**Affiliations:** I Divisao de Reumatologia, Faculdade de Medicina FMUSP, Universidade de Sao Paulo, Sao Paulo, SP, BR; II RDO Diagnosticos Medicos, Sao Paulo, SP, BR

**Keywords:** Granulomatosis with Polyangiitis, Vasculitis, Vitamin D, Respiratory Tract Infections, Disease Activity

## Abstract

**OBJECTIVES:**

To determine the possible association of serum 25-hydroxyvitamin D (25OHD) levels with disease activity and respiratory infection in granulomatosis with polyangiitis patients during two different periods: winter/spring and summer/autumn.

**METHODS:**

Thirty-two granulomatosis with polyangiitis patients were evaluated in the winter/spring, and the same patients (except 5) were evaluated in summer/autumn (n=27). The 25OHD levels were measured by radioimmunoassay. Disease activity was assessed by the Birmingham Vasculitis Activity Score Modified for Wegener’s Granulomatosis (BVAS/WG) and antineutrophil cytoplasmic antibody (ANCA) positivity. Respiratory infection was defined according the Centers for Disease Control and Prevention criteria.

**RESULTS:**

25OHD levels were lower among patients in winter/spring than in summer/autumn (32.31±13.10 *vs.* 38.98±10.97 ng/mL, *p*=0.04). Seven patients met the criteria for respiratory infection: 5 in winter/spring and 2 in summer/autumn. Patients with respiratory infection presented lower 25OHD levels than those without infection (25.15±11.70 *vs.* 36.73±12.08 ng/mL, *p*=0.02). A higher frequency of low vitamin D levels (25OHD<20 ng/mL) was observed in patients with respiratory infection (37.5% *vs.* 7.8, *p*=0.04). Serum 25OHD levels were comparable between patients with (BVAS/WG≥1 plus positive ANCA) and without disease activity (BVAS/WG=0 plus negative ANCA) (35.40±11.48 *vs*. 35.34±13.13 ng/mL, *p*=0.98).

**CONCLUSIONS:**

Lower 25OHD levels were associated with respiratory infection but not disease activity in granulomatosis with polyangiitis patients. Our data suggest that hypovitaminosis D could be an important risk factor for respiratory infection in granulomatosis with polyangiitis patients.

## INTRODUCTION

Granulomatosis with polyangiitis (GPA, previously known as Wegener’s granulomatosis) describes systemic vasculitis of small vessels that is strongly associated with antineutrophil cytoplasmic antibodies (ANCAs) directed against proteinase-3. GPA has many heterogeneous manifestations, especially in the upper and lower respiratory tracts and kidneys 1. The respiratory tract is frequently affected in GPA, with involvement of the upper airway or the development of pulmonary disease 2.

Patients with GPA have higher mortality during the first year of disease due to infection in most cases 3. In clinical practice, it can be very difficult to differentiate respiratory tract infection and disease activity in GPA. Airway infection and disease activity involving the respiratory tract are clinically very similar. Moreover, airway infection may elicit disease activity, and disease activity may exacerbate and perpetuate airway infection in GPA patients 4,5.

Vitamin D, in addition to acting on bone metabolism, acts as an immunomodulator, participating in innate and adaptive immune responses. 25-hydroxyvitamin D (25OHD) deficiency has been associated with disease activity in some autoimmune diseases, such as systemic lupus erythematosus and rheumatoid arthritis 6-8. 25OHD deficiency has also been associated with infection, particularly by respiratory viruses and mycobacteria 9-16.

Therefore, this study aimed to determine the possible association of 25OHD concentrations with disease activity and respiratory infections in patients with GPA during two different periods: winter/spring and summer/autumn. To the best of our knowledge, no studies have evaluated vitamin D serum levels in GPA patients and their association with disease activity or respiratory infection.

## MATERIALS AND METHODS

### Patient selection

**Figure 1:**
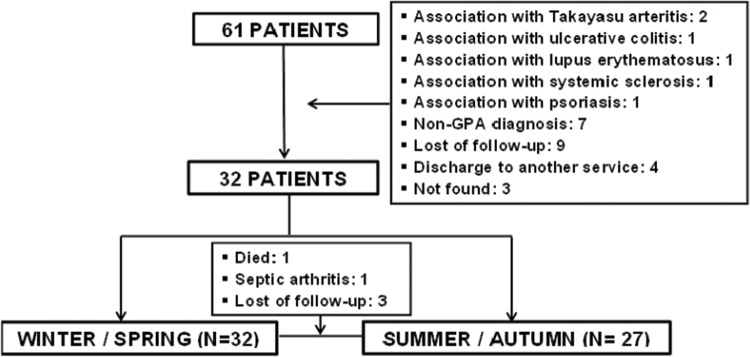
Selection of Granulomatosis with Polyangiitis (GPA) patients.

A total of 61 consecutive patients with GPA who regularly attended the outpatient Vasculitis Clinic of the Clinic Hospital of the University of São Paulo were screened from January 2011 to December 2013. All patients fulfilled the 1990 American College of Rheumatology (ACR) 17 and 2012 Revised International Chapel Hill Consensus criteria for the classification of GPA 18. Twenty-nine of these patients met the exclusion criteria due to the presence of other ANCA-associated diseases, autoimmune diseases, or concomitant non-respiratory infections. Therefore, 32 patients were evaluated (Figure 1). Demographic and clinical data, including race, age, disease duration, clinical manifestations, comorbidities, GPA treatment (glucocorticoid and immunosuppressive treatments) and vitamin D supplementation, were obtained through interviews with the patients and medical chart reviews. GPA was previously divided into clinical subgroups categorized by disease severity (localized, generalized and severe) 17.

The study was performed in accordance with the Declaration of Helsinki. All subjects provided written informed consent before inclusion in the study.

### Seasonal variations

Considering that vitamin D concentrations may be influenced by seasonal variations, serum 25OHD levels were measured during two periods of the year in each patient. Samples from 32 patients were evaluated in the winter/spring, and 27 samples from the same patients were evaluated in the summer/autumn, resulting in a total of 59 samples. Five patients were lost in the summer/autumn season: 1 patient died in another hospital from supposed pulmonary septic shock, 1 patient had a diagnosis of septic arthritis, and 3 patients were lost to follow-up.

### Disease activity and damage scores

GPA disease activity was assessed using the Birmingham Vasculitis Activity Score Modified for Wegener’s Granulomatosis (BVAS/WG), an established tool for clinical use and research that measures disease activity, vasculitis treatment outcomes, and the prognosis. The BVAS/WG measures activity according to 34 items that are categorized into 9 groups. The BVAS/WG values range from 0 to 63. Disease activity was defined as a BVAS/WG score ≥1, and remission was defined as a BVAS/WG score of 0 19. ANCA positivity was assessed using an indirect immunofluorescence method and was also used as a parameter of disease activity 20.

The Vasculitis Damage Index (VDI) was used to assess damage in all patients. The VDI is a validated checklist of 64 items divided into 11 organ-based systems and an ‘other’ category corresponding to potential treatment side effects. An item of damage was only recorded if it occurred after the onset of vasculitis and was considered permanent, defined as persistence for more than 3 months. In patients with established comorbidities prior to vasculitis, an item was only recorded if it had deteriorated significantly within at least 3 months since disease onset 21.

### Respiratory infection

Infection of the upper or lower airways was diagnosed according to the Centers for Disease Control and Prevention (CDC) criteria. Upper respiratory infection (pharyngitis, laryngitis, epiglottitis and sinusitis) and lower respiratory infection (pneumonia, bronchitis, tracheobronchitis, bronchiolitis and tracheitis) were defined based on clinical, laboratory, imaging and microbiological parameters 22.

### Laboratory parameters

Serum 25OHD levels were measured by radioimmunoassay (DiaSorin, Stillwater, MN, USA). Vitamin D deficiency was defined as 25OHD levels less than 20 ng/mL 23, with 25OHD concentrations sub-categorized as <20 ng/mL and ≥20 ng/mL.

C-reactive protein (CRP) levels and the erythrocyte sedimentation rate (ESR) were also evaluated because they may be elevated due to infection and/or disease activity. CRP was measured using the immunoturbidimetric method, and levels higher than 5 mg/L were considered abnormal. The ESR was measured by the Westergren test, with normal reference values in the first hour as follows: men <15 mm/h and women <20 mm/h. The white blood cell count (WBC) was measured in a local laboratory by an automated cell counter, with normal levels ranging from 4,000 to 11,000/mm^3^.

### Statistical analysis

Statistical analyses were conducted using the Statistical Package for the Social Sciences (SPSS for Windows, 21.0, SPSS Inc). The results are presented as the mean ± SD for continuous variables and as percentages for categorical variables. Quantitative variables were analyzed with Student’s T-test (normal distribution) or the Mann-Whitney (non-normal distribution) test. Differences between categorical variables were evaluated using the chi-squared or Fisher’s exact test. Statistical significance was indicated by *p* values less than 0.05. A receiver operator characteristic (ROC) curve was constructed using continuous serum 25OHD levels to identify a predictive value of 25OHD associated with respiratory infection.

## RESULTS

Fifty-three percent of GPA patients were women (n=17), and 65.6% (n=21) were white, with a mean age of 46.2±13.1 years. The mean age at diagnosis was 38.5±13.7 years, and the mean disease duration was 8.9±4.2 years. Most GPA patients had the generalized disease form (71.8%), and the localized form was observed in 28.2% of the patients.

Regarding the baseline clinical condition (winter/spring), 12 (37.5%) patients exhibited disease activity by the BVAS/WG criteria (BVAS/WG≥1), and the mean BVAS/WG was 1.37±2.49. Of these, 7 patients had upper airway involvement (58.3%), 5 had ocular involvement (41.6%), 4 had lower airway involvement (33.3%), 1 had neurological involvement (8.3%) and 1 had articular involvement (8.3%). The mean VDI was 5.5±2.3. ANCA positivity was observed in 59.4% of the patients in the winter/spring and in 70.4% of the patients in the summer/autumn. At baseline (winter/spring), 24 (75%) patients were using immunosuppressive agents, 24 (75%) were using trimethoprim-sulfamethoxazole, 16 (50%) were using prednisone and 3 (9.4%) were using rituximab.

Seven patients met the criteria for infection of the upper or lower airways: 5 in winter/spring and 2 in summer/autumn (Table 1). The same patient exhibited infection in winter/spring and subsequently in summer/autumn (patient #4 = patient #7). We observed 5 cases of tracheobronchitis, 1 case of pneumonia and 1 case of sinusitis. Table 1 shows the diagnostic criteria for respiratory infection. Two patients fulfilled the criteria for respiratory infection and the BVAS/WG criteria for respiratory activity simultaneously.

Table 2 shows the treatments applied in patients with disease activity and respiratory infection. All 7 patients with respiratory infection, except 1, were using immunosuppressive agents, and 5 were using prednisone (15.7±17.89 mg/day). No differences regarding prednisone, immunosuppressive agents or rituximab use were observed between GPA patients with and without infection. Moreover, no differences were observed relative to treatment in patients with and without disease activity (Table 2).

No difference in the frequency of respiratory infection relative to a particular season was observed (data not shown).

Of the 59 samples, 39 (66%) were obtained from patients who had received vitamin D supplementation (mean 800-7,000 IU/day). No difference was observed in 25OHD concentrations between patients with or without supplementation (34.87±12.51 *vs.* 36.31±12.81 ng/mL, *p*=0.68). However, 25OHD concentrations were significantly lower in patients in the winter/spring period than in the summer/autumn period (32.31±13.10 *vs.* 38.98±10.97 ng/mL, *p*=0.04).

No differences were observed in the data of GPA patients between the winter/spring season (n=32) and the summer/autumn (n=27) relative to the BVAS/WG (1.37±2.49 *vs.* 1.70±1.81, *p*=0.28), VDI (5.50±2.25 *vs.* 5.29±2.30, *p*=0.36), WBC (7,435±2,943 *vs.* 6,711±2,209/mm^3^, *p*=0.14), ESR (13.41±16.94 *vs.* 13.41±12.94 mm/h, *p*=0.49) and CRP level (6.00±10.72 *vs.* 10.49±23.71 mg/L, *p*=0.17).

No differences were observed regarding serum 25OHD levels and disease activity parameters relative to a BVAS/WG ≥1 *vs.* a BVAS/WG=0 (35.48±12.23 *vs.* 35.25±12.99 ng/mL, *p*=0.94), ANCA positivity *vs.* ANCA negativity (35.73±12.23 *vs.* 34.68±13.32 ng/mL, *p*=0.75), high CRP *vs*. normal CRP (37.06±13.68 *vs.* 34.67±12.13 ng/mL, *p*=0.51) and high ESR *vs.* normal ESR (35.06±11.63 *vs.* 35.51±13.10 ng/mL, *p*=0.89).

**Figure 2:**
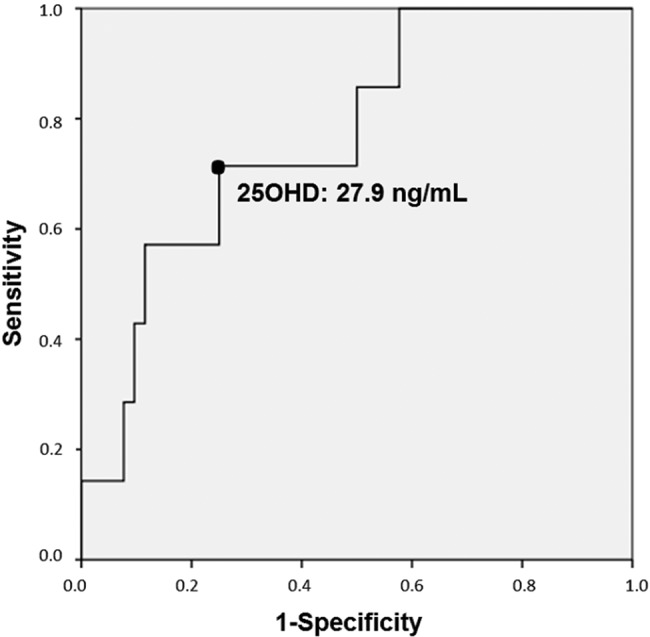
ROC curve analysis for serum 25OHD levels and respiratory infection in Granulomatosis with Polyangiitis (GPA) patients. 25OHD concentrations less than 27.9 ng/mL were predictor of respiratory infection with 71.4% sensitivity and 75% specificity.

In contrast, patients with a diagnosed respiratory infection had lower 25OHD concentrations than those without infection (25.15±11.70 *vs.* 36.73±12.08 ng/mL, *p*=0.02). Moreover, a higher frequency of low vitamin D levels (25OHD <20 ng/mL) was observed in patients with respiratory infection (37.5% *vs.* 7.8, *p*=0.04) (Table 3). ROC curve analysis indicated that a serum 25OHD level less than 27.9 ng/mL was associated with respiratory infection, with a sensitivity of 71.4%, a specificity of 75% and an area under the curve of 0.769 (Figure 2). For 25OHD levels less than 30 ng/mL, the sensitivity was 71.4%, and the specificity was 70.2% or respiratory infection.

## DISCUSSION

This study evaluated serum 25OHD levels in GPA patients and demonstrated for the first time that lower serum 25OHD levels were associated with respiratory infection in these patients. In fact, vitamin D deficiency has been linked to bacterial and viral infections, such as influenza, parainfluenza and respiratory syncytial virus 9-16,24,25. Our study confirmed and extended previous findings showing that 25OHD values lower than 20 ng/mL could be associated with respiratory infection. Epidemiologic studies have explored the association between seasonal variations in vitamin D levels and infections. In winter, lower serum vitamin D levels were associated with a higher incidence of infections, including septic shock 26, respiratory infection 27 and influenza 27,28.

Defense against infectious processes appears to be an extra-skeletal effect of vitamin D. Vitamin D increases the chemotaxis of inflammatory cells, enhances the phagocytosis of these cells, and stimulates the production of reactive oxygen species, leading to the destruction of pathogens. Vitamin D has also been associated with the direct production of cathelicidin, a peptide with bactericidal action 12,15,16,29. Regulation of the vitamin D receptor (VDR) is a mechanism used to defend against pathogens. However, some pathogens can evade the immune system by down-regulating VDR on the surfaces of monocytes and macrophages 30. VDR is present in most cells of the immune system, especially lymphocytes, neutrophils, macrophages and dendritic cells, and suppresses antigen presentation and the activation and recruitment of Th1 lymphocytes 15,31.

In our study, ROC curve analysis showed that serum 25OHD levels less than 27.9 ng/mL were associated with respiratory infection. Previous studies have shown that vitamin D status is associated with the risk of respiratory infection, especially in healthy individuals with levels below 20 ng/mL 4,5. Subclinical 25OHD deficiency was associated with severe lower respiratory tract infection in an Indian study 10, and clinical vitamin D deficiency was associated with a 13-fold increased risk of pneumonia in Ethiopian children 32. A Finnish study with young army recruits identified an association of 25OHD serum levels <16 ng/mL with acute respiratory infections and more days of absence from duty 33. In addition, Cannell et al. compiled epidemiological data regarding the association between seasonal variations in vitamin D levels and influenza and concluded that a lack of vitamin D during the winter may correspond to the infectivity of the influenza virus 28. A randomized, double-blind, placebo-controlled trial in schoolchildren demonstrated that vitamin D supplementation (1,200 U/day) significantly decreased the incidence of influenza infection by 42% in the winter 16.

Interestingly, in our study, no association between lower vitamin D levels and disease activity was observed, although the association between vitamin D levels and the immune system has been recognized in the literature. Vitamin D acts as an immunomodulator of the innate and acquired immune system, balances proinflammatory and anti-inflammatory cytokines, decreases the maturation of dendritic cells, reduces the proliferation of B lymphocytes, increases Th1 and Th17 responses and stimulates Treg cells 13. Poor vitamin D status is associated with the risk of rheumatologic diseases, including systemic lupus erythematous, rheumatoid arthritis and Behçet’s disease 7,34. However, no study has demonstrated an association of disease activity in ANCA-associated vasculitis with vitamin D serum levels and vitamin D supplementation.

As expected, our study identified lower serum 25OHD levels among subjects in the winter/spring period than in the summer/autumn period, reflecting the relationship between seasonality and vitamin D serum concentrations 16. Our patients were evaluated during two different periods, winter/spring and summer/autumn, but no difference between disease activity or infection was observed between seasons. Interestingly, in our study, no associations between infection or disease activity and CRP, ESR or WBC values were observed. Although these markers can be altered by certain conditions, laboratory parameters can also be affected by glucocorticoid and immunosuppressive treatments 35.

A limitation of this study is that our findings were restricted to more severe GPA disease because our patients predominantly had the generalized form of GPA with a high VDI, indicating greater severity and a higher risk of mortality 36. Moreover, we applied strict inclusion and exclusion criteria, and all patients, except for 5, were evaluated during two different periods. This study was also very strict in considering the CDC criteria for respiratory infection to avoid presumptive diagnoses of infection.

Lower 25OHD levels were associated with respiratory infection but not disease activity in GPA patients. Our data suggest that 25OHD hypovitaminosis could be an important risk factor for respiratory infection in GPA.

## AUTHOR CONTRIBUTIONS

Perez MO, Oliveira RM and Pereira RM were responsible for the study design. Perez MO and Pereira RM were responsible for the study conduct, data analysis and manuscript drafting. Perez MO, Oliveira RM, Caparbo VF, Levy-Neto M and Pereira RM were responsible for the data and laboratory collection, data interpretation, revision of the manuscript content and approval of the final version of the manuscript. Pereira RM takes responsibility for the integrity of the data analysis.

## Figures and Tables

**Table 1:** Respiratory infections in seven patients with Granulomatosis with Polyangiitis (GPA) and their respective BVAS/WG and serum 25OHD levels.

Patient	Infection	Infection Criteria	Season	BVAS	BVAS Criteria	25OHD ng/mL	Vitamin D supplementation
1	Pneumonia	Fever, cough, dyspnoea, leucocitosis, chest X-ray (pulmonary infiltrates), positive pleural fluid culture (*Acinetobacter baumanni*)	Winter/spring	0	-	18.52	No
2	Sinusitis	Fever, nasal discharge, headache, X-ray with evidence of infection	Winter/spring	2	Nasal crusts	25.19	Yes
3	Tracheobronchitis	Fever, cough, leucocitosis, chest X-ray without evidence of pneumonia	Winter/spring	2	Proptosis	19	Yes
4	Tracheobronchitis	Fever, cough, wheeze, increased pulmonar secretion, chest X-ray without evidence of pneumonia, positive bronchoalveolar lavage fluid culture (*Streptococcus viridans*)	Winter/spring	3	Neuropathy	37.65	Yes
5	Tracheobronchitis	Fever, coagh, leucocitosis, chest X-ray without evidence of pneumonia	Winter/spring	0	-	7.48	Yes
6	Tracheobronchitis	Fever, cough, wheeze, leucocitosis, chest X-ray without evidence of pneumonia	Summer/autumn	2	Hearing loss	41.55	Yes
7	Tracheobronchitis	Fever, cough, wheeze, increased pulmonar secretion, chest X-ray without evidence of pneumonia	Summer/autumn	3	Pulmonary	26.68	Yes

BVAS/WG: Birmingham Vasculitis Activity Score Modified for Wegener’s Granulomatosis.

**Table 2:** Current treatment in patients with Granulomatosis with Polyangiitis (GPA) with activity disease and respiratory infection.

	Disease Activity			Respiratory Infection		
	Activity (n=25)	No Activity (n=34)	*p*-values	Infection (n=7)	No Infection (n=52)	*p*-values
**Prednisone, n (%)**	15 (60)	11 (32.3)	0.06	5 (71.4)	20 (38.4)	0.12
**Immunosuppressive, n (%)**	21 (84)	19 (55.8)	0.09	6 (85.7)	34 (65.3)	0.41
**Rituximab, n (%)**	2 (8)	1 (2.9)	1.00	0	3 (5.7)	1.00

**Table 3:** Respiratory infection (presence or not), values of white blood cell count, C-reactive protein and erythrocyte sedimentation rate comparing patients with Granulomatosis with Polyangiitis (GPA) with serum levels of 25OHD ≥ 20 ng/mL vs. < 20 ng/mL.

	25OHD ≥ 20 ng/mL (n=51)	25OHD < 20 ng/mL (n=8)	*p*-values
**Infection**			0.04
No	47 (92.2%)	5 (62.5%)	
Yes	4 (7.8%)	3 (37.5%)	
**WBC**			0.13
High	3 (5.9%)	2 (25%)	
Normal	48 (94.1%)	6 (75%)	
**CRP**			1.00
High	15 (29.4%)	2 (25%)	
Normal	36 (70.6%)	6 (75%)	
**ESR**			0.70
High	18 (35.3%)	2 (25%)	
Normal	33 (64.7%)	6 (75%)	

Respiratory infection by Centers for Disease Control and Prevention (CDC) criteria.

WBC: White blood cell count (normal values: 4,000 - 11,000/mm^3^).

CRP: C-reactive protein (high: >5 mg/L).

ESR: Erythrocyte sedimentation rate (high: >15mm for men and >20mm for women).
